# Characterization of mouse orofacial pain and the effects of lesioning TRPV1-expressing neurons on operant behavior

**DOI:** 10.1186/1744-8069-4-43

**Published:** 2008-10-01

**Authors:** John K Neubert, Christopher King, Wendi Malphurs, Fong Wong, James P Weaver, Alan C Jenkins, Heather L Rossi, Robert M Caudle

**Affiliations:** 1 Department of Orthodontics, College of Dentistry, University of Florida, Gainesville, FL, USA; 2 Department of Oral Surgery, College of Dentistry, University of Florida, Gainesville, FL, USA; 3 Department of Prosthodontics, College of Dentistry, University of Florida, Gainesville, FL, USA; 4 Department of Neuroscience, College of Medicine, University of Florida, Gainesville, FL, USA; 5 Evelyn F. and William L. McKnight Brain Institute, University of Florida, Gainesville, FL, USA

## Abstract

**Background:**

Rodent models of orofacial pain typically use methods adapted from manipulations to hind paw; however, limitations of these models include animal restraint and subjective assessments of behavior by the experimenter. In contrast to these methods, assessment of operant responses to painful stimuli has been shown to overcome these limitations and expand the breadth of interpretation of the behavioral responses. In the current study, we used an operant model based on a reward-conflict paradigm to assess nociceptive responses in three strains of mice (SKH1-Hr^hr^, C57BL/6J, TRPV1 knockout). We previously validated this operant model in rats and hypothesized in this study that wild-type mice would demonstrate a similar thermal stimulus-dependent response and similar operant pain behaviors. Additionally, we evaluated the effects on operant behaviors of mice manipulated genetically (e.g., TRPV1 k.o.) or pharmacologically with resiniferatoxin (RTX), a lesioning agent for TRPV1-expressing neurons. During the reward-conflict task, mice accessed a sweetened milk reward solution by voluntarily position their face against a neutral or heated thermode (37–55°C).

**Results:**

As the temperature of the thermal stimulus became noxiously hot, reward licking events in SKH1-Hr^hr ^and C57BL/6J mice declined while licking events in TRPV1 k.o. mice were insensitive to noxious heat within the activation range of TRPV1 (37–52°C). All three strains displayed nocifensive behaviors at 55°C, as indicated by a significant decrease in reward licking events. Induction of neurogenic inflammation by topical application of capsaicin reduced licking events in SKH1-Hr^hr ^mice, and morphine rescued this response. Again, these results parallel what we previously documented using rats in this operant system. Following intracisternal treatment with RTX, C57BL/6J mice demonstrated a block of noxious heat at both 48 and 55°C. RTX-treated TRPV1 k.o. mice and all vehicle-treated mice displayed similar reward licking events as compared to the pre-treatment baseline levels. Both TRPV1 k.o. and RTX-treated C57BL/6J had complete abolishment of eye-wipe responses following corneal application of capsaicin.

**Conclusion:**

Taken together, these results indicate the benefits of using the operant test system to investigate pain sensitivity in mice. This ability provides an essential step in the development of new treatments for patients suffering from orofacial pain disorders.

## Background

Orofacial pain encompasses a multitude of disorders, including temporomandibular disorders (TMDs), trigeminal neuralgia, headaches, and myofascial pain, and affects an estimated 20% of the U.S. population [1]. Some distinct orofacial pain disorders, such as trigeminal neuralgia and migraine headache have no analogous counterparts in other parts of the body (e.g., below the neck). While systems underlying the orofacial pain pathway appear to be similar to those elsewhere in the body, using the same receptors, neurocircuitry (e.g., c-fibers, Aδ fibers), and neurotransmitters (e.g., substance P, glutamate), it is unclear, however, whether trigeminal pain processing is mediated differently. Therefore, investigating mechanisms affecting the trigeminal system may provide unique treatment options for those suffering from orofacial pain.

To this end, most pain models in rodents, which have typically targeted the hindpaw, have been adapted to the orofacial region. These models involve: the induction of inflammation [2-4]; production nerve injuries [5-7]; neurogenic inflammatory agents (e.g., capsaicin) [8,9]; formalin [10,11]; Complete Freund's adjuvant (CFA) [12]. One major limitation in these studies, though, involves the assessment and interpretation of the behavioral responses. Testing in the facial region provides a unique set of hurdles, as many of the behavioral assays require restraining the animals and are often associated with a great degree of time and training on the part of the experimenter. For example, assessment of mechanical sensitivity typically involves holding an animal while applying a von Frey filament to the surface of the skin, but there are visual anticipatory cues to contend with if the animal's eyes are unshielded. Additionally, what experimenter defines as a "painful response" can be as varied as a head withdrawal to freezing, whereby an animal becomes completely unresponsive, which is indistinguishable from a fear response. The investigator can overcome some of these limitations by using a "hands-free" operant design to assess facial pain behaviors.

Operant systems utilizes a reward-conflict paradigm, whereby an animal can decide between receiving a reward or escaping an aversive stimulus [13,14]. In this case, the animal controls the amount of nociceptive stimulation and can modify its behavior based on cerebral processing [15,16]. We recently made great strides in bridging the gap in orofacial thermal testing with the development of a novel operant orofacial testing paradigm. We validated and characterized an operant thermal assessment paradigm in rats with both hot [17] and more recently cold [18] stimuli, and evaluated this system using a number of pain models, including capsaicin-induced neurogenic inflammation [19], carrageenan-induced inflammation [17], and menthol-induced sensitization [18]. However, thus far our assessment of thermal processing has been limited to rats.

The use of mice to evaluate nociception has gained momentum, as most pain induction methods and behavioral assays traditionally applied to rats can be easily scaled for application in mice. The advent of transgenic technology in the mouse has allowed an unprecedented look into basic mechanisms relating to pain modulation. There are many major classes of pain related molecules that have been targeted using transgenic manipulations, including neurotrophins, peripheral factors, opioids, intracellular signal transducers, and non-opioid systems [20]. However, behavioral assessments of pain in mice still primarily use reflex-based methods. Operant tests for mice such as the conditioned place preference task (CPP) have been used extensively in field of addiction research [21] and, to some degree, in the field of pain research [22]. Recent work in transient receptor potential channel melastatin 8 (TRPM8) knockout mice has also used preference tasks to evaluate the cold processing in the hindpaw [23,24]. However, these assessments focus on pain induction in the hindpaw. Therefore, we sought to adapt our operant orofacial assay, previously used with rats, for use with mice. Although the nociceptive pathways are generally similar in rats and mice, some behaviors seen in rats are not reliably noted in mice, such as the flinching behavior following injection of formalin [25]. Other differences may exist between the two species that could affect performance on our behavioral assay, such as level of attentiveness, tendency to engage in exploratory behavior, and the rewarding potential of the milk solution.

The current study had several goals. The first goal of this study was to characterize the operant behavior of mice exhibited in the presence of a range of neutral to hot stimuli and evaluate if their behavior is strongly influence by the nociceptive potential of the stimulus, as is true of rats [17]. Additionally, we wanted to evaluate the responses of wild-type (w.t.) mice following induction of pain with and without analgesic intervention. Once we established this behavioral profile in genetically w.t. mice, our second goal was to characterize operant behavior of TRPV1 k.o. mice to thermal stimuli. Original behavioral characterization of this strain has focused on withdrawal latency and innate nocifensive behaviors, but no studies have assessed the behavioral phenotype of this knock out in an operant context. In addition, it is becoming apparent that the encoding of thermal sensations relies on co-expression and interaction among the thermal TRP channels, as well as others. Combining genetic manipulations with pharmacological manipulations can provide insights to the how changes in these channels influence behavior towards thermal stimuli. Thus, our final goal was to compare the behavior exhibited by TRPV1 k.o. mice to w.t. C57BL/6J counterparts treated with resiniferatoxin (RTX), a potent TRPV1 agonist used for lesioning agent TRPV1-expressing neurons. This work will serve to enhance the understanding of how nociceptive processes in the trigeminal system are integrated and will be critical for the advancement of the field of orofacial pain research for the transition into clinical treatments.

## Materials and methods

### General

Hairless *SKH1-Hr*^*hr*^, TRPV1 k.o., and age-matched wild-type controls (C57BL/6J) mice were obtained from Jackson Laboratories (Bar Harbor, ME). TRPV1 k.o. and C57BL/6J mice were lightly anesthetized with isoflurane (1–2.5%, inhalation) and their fur was bilaterally removed from the orofacial region using depilatory cream at least 1 day prior to behavioral testing. All mice were food fasted for 15 hrs prior to each facial testing session and provided with standard food chow at the completion of testing. Mice were tested at the same time of the day and weighed weekly to monitor health. A recovery day was included between the testing sessions to minimize nutritional deficits due to fasting. Water was also made available *ad libitum *before and after testing sessions. The mice were placed into the behavioral procedure room 30 min prior to testing and allowed to acclimate to the temperature and ambient noise of the room. Animal testing procedures and general handling complied with the ethical guidelines and standards established by the Institutional Animal Care & Use Committee at the University of Florida and all procedures complied with the Guide for Care and Use of Laboratory Animals [26].

### General activity assessment – rearing behavior

A potential confounding factor for completing an operant behavioral test (*see below*) includes strain differences in general activity or due to a specific treatment. For example, if a drug produces significant sedation, one would expect an animal to perform poorly on the operant task, which could provide a false negative result in terms of analgesic-potency for that drug. While no analgesic drug were evaluated in the current study in terms of altered rearing activity, we modified our rat rearing chamber design [27] to accommodate the testing of the mice to measure rearing activity as an assessment of general behavior. An acrylic cylinder (11 cm diameter × 17.6 cm height) was constructed with aluminum sheets placed both on the floor and on 6.3-cm above the floor. The metal siding was connected to a DC power supply and, in series, to a multi-channel data acquisition module (DATAQ Instruments, Inc) and the floor served as the ground for the circuit. Unrestrained mice were placed separately into each cylinder and the data acquisition system was activated. During a rearing event, the animal would place its front paw on the metal side of the cylinder and this would complete an electrical circuit that registered in the computer. Each session lasted 10 min and the number of contacts, duration of contacts, and the duration of each contact was determined. Animals were tested 7 sessions at the same time of the day (afternoon) over a period of two and a half weeks.

### Thermal testing

To change the effective size of the test box, we modified our existing rat operant test chamber, which consisted of a 20.3 cm W × 20.3 cm D × 16.2 cm H acrylic box, by placing an acrylic insert (7 cm W × 7 cm D × 8 cm H) onto an elevated platform (6 cm off the floor) to effectively reduce the size of the internal dimensions to better accommodate mice. The existing opening (4 × 6 cm) was which was lined with grounded metal (aluminum) tubing was also reduced to a 1" × 1" opening using an acrylic face plate. This again served as a *stimulus thermode *when connected to a heated circulating water pump (Model RTE-7 D+, Thermo Electron) [17]. The stimulus temperature was adjusted from neutral to very hot (37 to 55°C) and the stimulus thermode temperature was verified for each experiment using a contact thermometer (Fluke, Model 54II).

**Figure 1:**
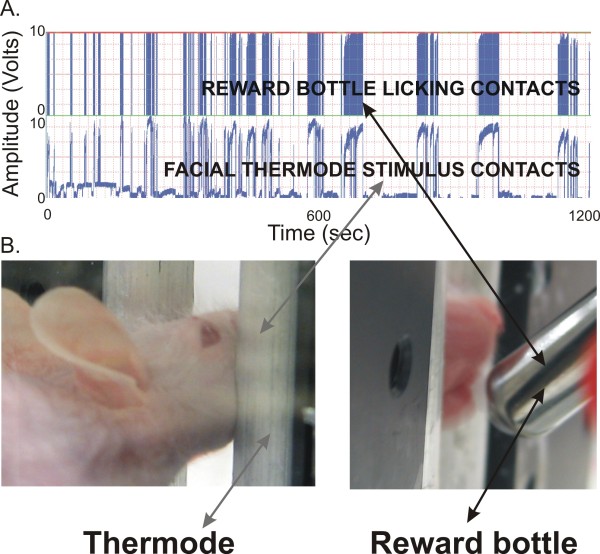
**Mouse facial thermal operant assay**. (A) Reward licking contacts (*upper trace*) and facial thermode stimulus contacts (*lower trace*) acquired in a typical 20 min test session at 37°C. (B) Example of a hairless *SKH1-Hr*^*hr *^mouse completing the task by contacting the thermode (*left*) while simultaneously licking from the reward bottle (*right*).

Unrestrained animals were placed separately in each testing cage insert and the reward bottle containing diluted (1:2 with water) sweetened condensed milk solution (Nestle, room temperature) was positioned in proximity to the cage such that the animal will be allowed access to the reward bottle when simultaneously contacting the thermode with its face (Figure 1; Additional file 1). The metal spout on the watering bottle was connected to a DC power supply and, in series, to a multi-channel data acquisition module (WinDaq Data Acq DI-710-UH, DATAQ Instruments, Inc). When the mouse completed the task and drank from the bottle, the animal's tongue contacted the metal spout on the water bottle, completing an electrical circuit (Figure 1A, upper trace). The closed circuit was registered in the computer and each spout contact was recorded as a "licking" event. The threshold for detection was set to eliminate noise and minimize artifacts. A separate circuit was established from the aluminum thermode to the animal by grounding the floor with an aluminum sheet for recording of "facial contact" events (Figure 1A, lower trace). The latter circuit is necessary to determine if the animal is discouraged by the thermal stimulus. The investigator monitored online data acquisition to ensure that each recorded licking event from the first circuit corresponded to a recorded facial contact on the tubing (the second circuit) to ensure that the animal did not access the reward while avoiding the thermode, and it minimized false-positive recordings of licks. A complete session lasted 20 min. During offline data analysis, the threshold for detection of the licking contacts was set above background noise, to minimize false positive event registration and events typically registered as > 5.0 V. An event was registered when the signal went above threshold and ended when the signal dropped below threshold. Data analyses were completed using custom-written routines (generously provided by Dr. Charles Widmer, University of Florida) in LabView Express (National Instruments Corporation) and Excel (Microsoft). The total number of reward licking events was determined for each mouse and compared across experimental conditions. Room temperature was maintained at 22 ± 1°C for all behavioral tests.

Animals were first trained to drink milk while contacting metal tubing in a training box that was of similar dimensions to the thermal testing box. The training boxes do not have connections to the water bath and hence the metal contact was equal to the room temperature. This lead-in training period was necessary to acquaint the animals with the task of locating the reward bottle. Animals were considered trained when they achieved a total of ≥ 1000 licks in the training box. To evaluate the effects of food fasting, a subset of animals were tested first without fasting for three training sessions and then with overnight fasting for three sessions over a period of 2 weeks.

### Induction of neurogenic inflammation in the face

We induced pain and nociceptive sensitization in the orofacial region using the neurogenic inflammatory agent, capsaicin as described previously by our group [19]. Briefly, capsaicin cream (0.035%, Chattem, INC, Chattanooga, TN) was liberally applied to facial region of anesthetized (isoflurane, inhalation, 2%) SKH1-Hr^hr ^mice (N = 8) and left on for 5 min. The capsaicin was then removed using a moist paper towel, followed by a 70% EtOH wipe and animals were tested 30 min post-capsaicin application at 47°C. One set of animals (N = 5) was treated in the same manner with capsaicin, but also was administered morphine (s.c., 0.5 mg/kg, 100 μl) between the scapulae immediately post-capsaicin application (30 min prior to testing). Note that the 0.5 mg/kg dose was chosen to minimize side effects such as sedation, as this may significantly impact completion of the operant task [27].

To evaluate the effects of morphine on feeding and reward behavior at a non-noxious stimulus temperature, we tested a separate group of SKH1-Hr^hr ^mice (N = 10) at 37°C. Five animals received morphine (s.c., 0.5 mg/kg, 100 μl) and five received vehicle (dH_2_O, s.c., 100 μl) and then were evaluated 30' post-injection using the operant system. The treatments were crossed-over for a second session 1-week later and the treatment groups combined for final analysis.

### Resiniferatoxin (RTX) administration

Resiniferatoxin (RTX) is an ultrapotent TRPV1-specific agonist that we have used as a molecular neurosurgical agent to selectively lesion TRPV1-positive afferent neurons [28,29]. Central administration of RTX or vehicle (0.25% Tween 80 in phosphate buffered saline (PBS), 0.05% ascorbic acid) was achieved by injection into the cisterna magna in TRPV1 k.o. (N = 5/treatment) and their wild-type CB57BL/6 control mice (N = 5/treatment). The skin overlying the occipital and cervical regions of the head and neck was disinfected with Betadine. A 30-gauge needle tip connected to Hamilton syringe via PE-10 tubing was carefully directed to touch the mid- to lower portion of the occiput. Note that contacting the bony surface provided distinct tactile feedback as the tip was sequentially moved caudally until the needle punctured the atlanto-occipital membrane overlying the cisterna magna. RTX (100 μg, 1 μl) or vehicle (1 μl) was injected slowly over 10 seconds to allow the solutions to mix with the cerebrospinal fluid (CSF). Animals recovered for 1-week prior to behavioral testing.

A capsaicin eye-wipe test was completed [30] prior to and following the injections to assess TRPV1 functionality. Briefly, a 0.1% capsaicin solution (20 μl) was placed onto the cornea of the eye of gently restrained animals and the number of eye wipes was counted for 1 min. A negative response (no wipes) verified RTX-lesioning of TRPV1 afferent neurons and only animals that were negative were used in the final operant comparisons. Capsaicin eye-wipe was also completed on TRPV1 k.o. mice as a negative control for comparison.

### Statistical analysis

Statistical analyses were completed (SPSS Statistical software, SPSS Inc) including Student's T-tests and ANOVAs to evaluate the effects of temperature or treatment on the reward licking outcome measure, and a general linear model for multivariate analysis was used to assess the effects of time and strain on rearing behavior. When significant differences were found, post-hoc comparisons were made using the Tukey HSD. *P < 0.05 was considered significant in all instances.

## Results

### General activity and rearing behavior

We used a rearing chamber with automated data acquisition to assess the overall activity of three strains of mice. The number of rearing events significantly decreased for *SKH1-Hr*^*hr *^and CB57Bl/6J strains over the two-week test period, but not TRPV1 k.o. mice (Table 1A). The duration of rearing events was not significantly different for any of the strains (Table 1B). When comparing the number of rearing events between the strains over the 7 sessions, we found that there was a significant time effect (F_6,31 _= 5.65, P < 0.001), but not a significant time*strain interaction (F_12,64 _= 1.59, P = 0.116). This was also true of rearing duration: time (F_6,23 _= 3.99, P = 0.007); time*strain (F_12,48 _= 0.89, P = 0.568). Tests of within-subjects effects demonstrated a significant time effect (F_6,216 _= 5.82, P < 0.001), but not a significant time*strain interaction (F_12,216 _= 1.74, P = 0.059). This was again similar for the duration of rearing: time (F_6,168 _= 3.18, P = 0.006); time*strain (F_12,168 _= 0.79, P = 0.655). Tests of between-subjects effects on number of events demonstrated a significant difference between the strains (F_2,36 _= 3.92, P = 0.029), with post-hoc tests revealing that the hairless *SKH1-Hr*^*hr *^mice had significantly greater rearing activity as compared to the C57BL/6J strain. The duration of rearing followed in a similar fashion (F_2,28 _= 4.18, P = 0.026). For both measures, the TRPV1 had an intermediate level of activity, but were not significantly different from either of the other two strains.

**Table 1:** Rearing behavior.

		**STRAIN (Mean ± SEM)**	
**A. EVENTS**			
*Session*	*Hairless*	*C57BL/6J*	*TRPV1 k.o.*
*1*	103.3 ± 9.6	50.6 ± 5.3	53.1 ± 3.8
*2*	77.8 ± 12.8	50.6 ± 5.5	71.6 ± 10.4
*3*	*58.7 ± 6.4	34.1 ± 5.1	54.8 ± 14.1
*4*	70.1 ± 8.0	44.6 ± 7.8	48.3 ± 6.8
*5*	72.5 ± 10.5	31.8 ± 3.2	40.1 ± 9.7
*6*	*58.0 ± 8.2	33.0 ± 4.7	52.4 ± 8.3
*7*	*51.0 ± 5.8	28.9 ± 5.2	37.5 ± 6.3
Significance level	(F_6,176 _= 3.5, P = 0.003)	(F_6,69 _= 2.9, P = 0.014)	(F_6,60 _= 1.5, P = 0.201)

**B. DURATION**			
*Session*	*Hairless*	*C57BL/6J*	*TRPV1 k.o.*

*1*	57.4 ± 6.1	38.6 ± 5.0	48.5 ± 5.7
*2*	59.5 ± 8.5	38.5 ± 3.1	56.0 ± 7.2
*3*	45.5 ± 7.0	31.3 ± 4.2	41.7 ± 7.1
*4*	52.1 ± 7.3	41.6 ± 5.3	54.6 ± 9.1
*5*	52.1 ± 7.3	34.6 ± 3.2	36.3 ± 8.3
6	46.9 ± 7.5	30.2 ± 2.8	39.4 ± 7.1
7	51.2 ± 7.5	26.0 ± 4.0	37.2 ± 7.9
Significance level	(F_6,168 _= 0.46, P = 0.837)	(F_6,69 _= 1.9, P = 0.093)	(F_6,60 _= 1.2, P = 0.324)

General activity of the three strains of mice (C57BL/6J, TRPV1 k.o., hairless SKH1-HRHR) was assessed by determining the number of rearing events and duration of the rearing events over the 7-sessions. There was a significant decrease in rearing events for the hairless and C57BL/6J strains (ANOVA, *P < 0.05).

**Figure 2:**
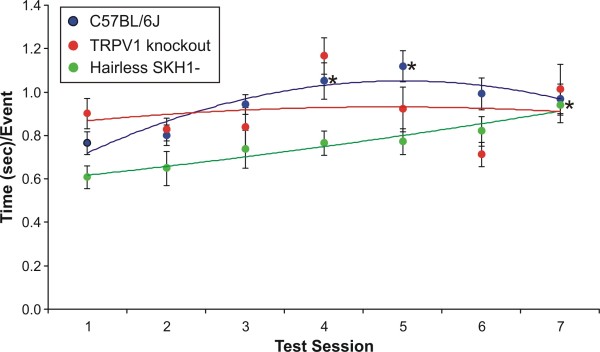
**Rearing behavior**. General activity of the three strains of mice (C57BL/6J, TRPV1 k.o., hairless *SKH1-Hr*^*hr*^) was assessed by monitoring rearing activity. The amount of time (sec) per rearing event was calculated and plotted over several test sessions, completed over the course of 2.5 weeks. There was a significant increase in the time/event ratio for the hairless and C57BL/6J strains over time; however, there were no between strain differences at the later time points, indicating a similar level of accommodation for the strains. *denotes values significantly higher than the first session (P < 0.05).

When we evaluated the average rearing (e.g., duration/event ratios) across seven testing sessions, there was a significant effect of time on this outcome (F_6,23 _= 4.06, P = 0.006), but not a significant time*strain effect (F_12,48 _= 1.40, P = 0.200). The hairless (F_6,168 _= 3.13, P = 0.006) and CB57Bl/6J (F_6,69 _= 3.8, P = 0.003) mice had significantly higher ratios over time, but each strain had values that were not significantly different from each other by the seventh session (Figure 2). The within-subject effects of time were significant (F_6,168 _= 5.41, P < 0.001), but time*strain effects were not significant (F_12,168 _= 1.36, P = 0.191). There was no significant between-subjects effects for strain (F_2,28 _= 1.94, P = 0.162). Overall, the decrease in the rearing events and duration across testing sessions indicate that all three strains habituate to the testing environment. The rearing duration/event ratio indicates that while there may initially be differences in activity level between strains, these differences are no longer significant with repeated testing.

### 2. Effects of thermal stimuli on operant behavior (Figure 3)

**Figure 3:**
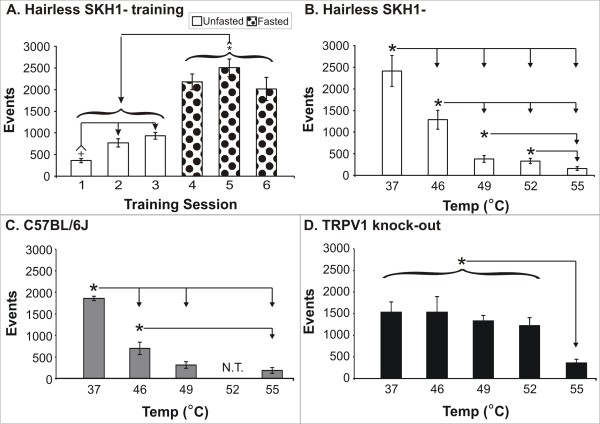
**Operant reward licking events for three different strains of mice**. Hairless mice significantly increased their reward licking events (A) when fasted overnight (N = 13–27/session). Unfasted animals had significantly lower (**+**P < 0.05) licking events in session 1 as compared to session 2, but there was no difference between session 2 and 3, and overall the fasted sessions produced significantly higher performance as compared to the unfasted sessions. Hairless (B) and C57/BL6 (C) mice demonstrated a stimulus response such that increasing temperatures significantly reduced the number of reward licking events, indicating aversion to the more noxious stimuli. The TRPV1 knock-out mice (D) were relatively insensitive to temperatures ≤ 52°C, as their responses in the noxious heat range of 46–52°C produced responses similar to baseline 37°C testing conditions. These knock-out mice only demonstrated a significant decrease in reward licking events when they were tested at the highest stimulus temperature of 55°C. A total of N = 10 hairless, N = 5 CB57BL6, and N = 5 TRPV1 knock-out mice were used to generate these data. N.T. = not tested. *Significantly higher (P < 0.05). Note that there were no significant differences between male and female mice when comparing the number of licking events during the unfasted and fasted sessions, therefore their data was pooled.

The mice completed the reward-conflict task in a fashion similar to rats [17], whereby they would contact a thermode with their face to access a reward bottle, generating an electrical signal that was acquired for analysis (*see Figure *1A, B*above*). There was a significant increase in reward licking events when animals were fasted overnight (Figure 3A). During the unfasted sessions, there was a significant effect of testing day (F_2,72 _= 16.95, P < 0.001). The first session had significantly lower licks than the second and third sessions. This initial increase may be due to animals learning the task; however, the outcomes during the fasted sessions were relatively stable, as there was not a significant effect due to test session on licks for the fasted sessions, indicating that the behavior was relatively stable with fasting and not indicative of further learning-related increases.

There was a significant effect of temperature on licking behavior for all three strains: *SKH1-Hr*^*hr *^(F_4,49 _= 23.61, P < 0.0001), C57BL/6J (F_3,19 _= 40.26, P < 0.0001), TRPV1 k.o. (F_3,19 _= 5.89, P = 0.007). Post-hoc analyses revealed that the *SKH1-Hr*^*hr *^and C57BL/6J mice displayed the normal operant pain behavior of significantly decreasing reward-licking performance as the temperature increases into the noxious hot range (Figure 3B, C). The TRPV1 k.o. mice (Figure 3D); however, exhibited a rather flat response with stimulus temperatures of 37–52°C, with no significant decrease in reward licking across this range. There was a significant decrease in reward licking only at the highest temperature tested, 55°C.

**Figure 4:**
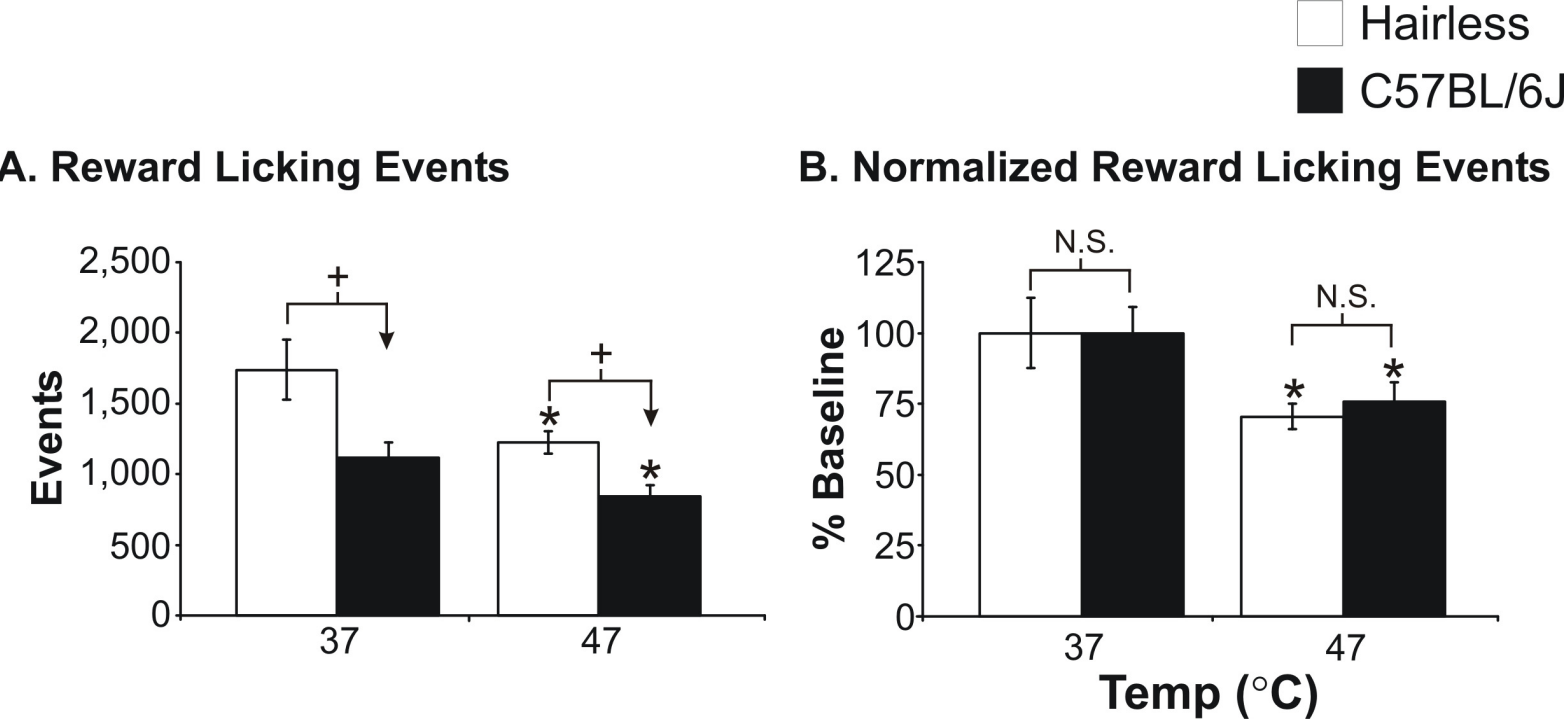
**Operant reward licking comparison of hairless *SKH1-Hr*^*hr *^versus C57BL/6J wild-type mice**. The hairless *SKH1-Hr*^*hr *^mice had significantly greater (^+^P < 0.05) licking events as compared to the C57BL/6J mice at both neutral (37°C) and hot (47°C) stimulus temperatures (A). When the data was normalized to the neutral temperature (37°C) as a percent baseline for each strain, the differences between the two strains were not significant (N.S.). For both strains, there was a significant decrease (*P < 0.05) in both raw and normalized licking events at 47°C as compared to 37°C, indicating an aversive response elicited by the noxious stimulus temperature.

We used the *SKH1-Hr*^*hr *^strain extensively in this and other studies; however, we realize that this is not the typical strain used to study pain, unlike the C57BL/6J or 129 strains. Therefore as part of the thermal-stimuli comparison, we evaluated the response of the *SKH1-Hr*^*hr *^strain as compared to the C57BL/6J strain, relative to their respective baseline 37°C results (Figure 4). We found that there are raw value differences when testing different strains at these stimulus temperatures (Figure 4A); however, the direction and magnitude of the response to nociceptive stimuli was the same between the strains, as indicated when licking events are normalized to 37°C (Figure 4B). The hairless strains tend to consume more at each temperature, but the relative change in performance with a painful stimulus is similar for both strains of mice.

### 3. Effects of neurogenic inflammation on operant behavior

**Figure 5:**
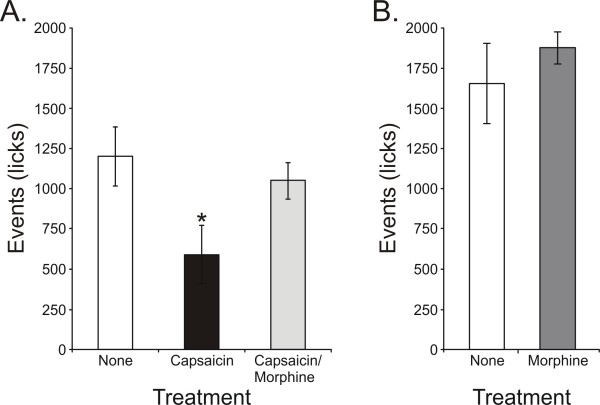
**Effects of neurogenic inflammation and morphine on operant reward licking behavior in hairless *SKH1-Hr*^*hr *^mice**. Hairless *SKH1-Hr*^*hr *^mice treated with capsaicin cream (0.035%, topical, 5') had significantly lower reward licking events (*P < 0.05) as compared to baseline (A) at 47°C. Morphine (0.5 mg/kg) given concurrently with the capsaicin-treatment provided a significant antihyperalgesic effect under similar testing conditions, as the reward licking counts were significantly higher than the capsaicin-alone group (A). A different set of animals (N = 10) was tested with either morphine (0.5 mg/kg, s.c.) or water (100 μl, s.c.) at 37°C in order to assess the effects of morphine on feeding and reward behavior in the absence of a painful stimulus (B). There were no significant differences between the two treatment groups at this neutral stimulus temperature.

We previously tested the pain sensitivity of rats following capsaicin-induced neurogenic inflammation and evaluated the effects of a low-dose of morphine on this model of thermal hyperalgesia [19]. Here we evaluated the effects of capsaicin and capsaicin/morphine on operant licking reward behavior in the *SKH1-Hr*^*hr *^strain, tested at 47°C (Figure 5). We found that there was a significant treatment effect on reward licking when animals were treated with capsaicin and capsaicin/morphine (F_2,22 _= 7.70, P = 0.003). Post-hoc analyses reveal that the capsaicin-treated animals had significantly lower licking events as compared the baseline session (no treatment) and this hyperalgesic response was reversed with treatment of morphine. The capsaicin/morphine-treated animals had licking events similar to baseline levels, but significantly higher than the capsaicin-treated animals.

### 4. Effects of intracisternal RTX on w.t. mice and TRPV1-k.o. mice

**Figure 6:**
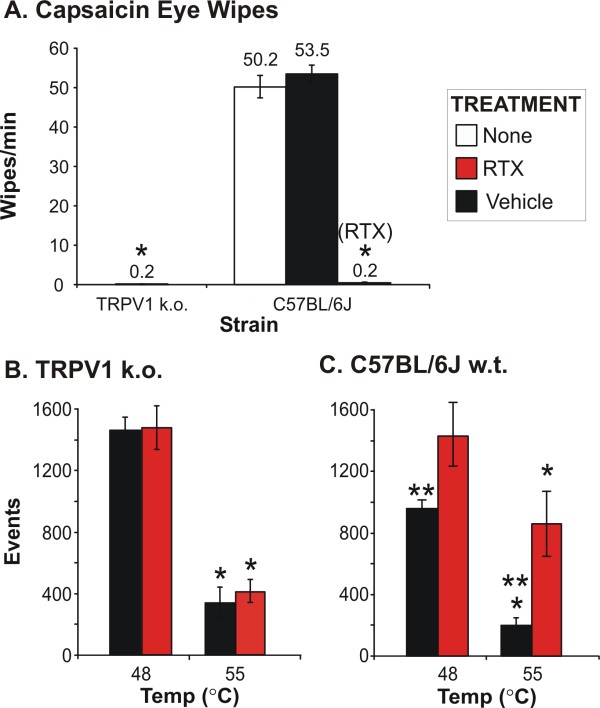
**Intracisternal (ICM) injection of RTX blocked heat pain in the face for wild-type, but not TRPV1 knock-out mice**. Animals treated 1-week previously with either RTX (100 ng, 1 μl) or vehicle (0.25% Tween 80 in PBS) were tested using the capsaicin eye-wipe assay and using the operant orofacial device at 48 and 55°C. TRPV1 k.o. mice and RTX-treated C57BL/6J mice were completely insensitive to capsaicin (0.1%, 20 μl, corneal application), while vehicle-treated C57BL/6J had a response similar to non-treated animals (A). TRPV1 k.o. mice treated with RTX responded similarly to vehicle treated k.o. mice at both 48 and 55°C (B). C57BL/6J mice treated with RTX were not sensitive to the 48°C stimuli (B) and behaved like the TRPV1 k.o. mice. Interestingly, the RTX-treated C57BL/6J mice demonstrated a significant increase in response at 55°C, with licking events increased even above the TRPV1 k.o. mice. * = significantly lower (P < 0.05), compared to baseline; ** = significantly lower compared to RTX-treated animals.

We wanted to compare the behavioral effects of pharmacological removal of TRPV1 using RTX with the genetic removal of TRPV1. Not surprising, TRPV1 k.o. mice were completely insensitive to corneal application of capsaicin, as they had a significantly lower eye-wipe response as cozmpared to untreated w.t. C57BL/6J mice (Figure 6A). In fact, these animals were completely unresponsive, with the exception of an animal or two removing the liquid with a few wipes. The w.t. C57BL/6J mice treated with i.c.m. RTX also had a significantly lower response compared to naïve and vehicle (i.c.m.) w.t. animals (Figure 6A). There was no difference in the response between TRPV1 k.o. and RTX-treated C57BL/6J mice.

For the operant testing, we found at 48°C, the TRPV1-k.o. mice had significantly higher licking events as compared to the C57BL/6J mice, indicating that the k.o. mice were insensitive to the hot stimulus that is within the activation range of TRPV1. When treated with RTX, the C57BL/6J w.t. mice had significantly higher licks as compared to vehicle treated animals, on par with the TRPV1-k.o. mice (Figure 6C). When tested at 55°C, all of the TRPV1-k.o. mice (vehicle, RTX) had significantly lower licks as compared to baseline (37°C, Figure 6B). In TRPV1 k.o. mice, no differences in licking behavior was observed at any of the temperatures between mice treated with RTX as compared to vehicle treated mice (Figure 6B). The RTX-treated CB57BL/6J mice had significantly higher licks as compared to the vehicle treated animals when tested in the noxious heat range. Taken together these results confirm that RTX is specific for TRPV1 and that the effects of pharmacological lesion are not the same as genetic removal of TRPV1. These results indicate that another high-threshold receptor is likely linked to TRPV1. Reduction of this other receptor can reduce sensitivity to high thermal stimuli in the w.t. mice, thus, producing a significantly higher lick output. Note that naïve animals in both the TRPV1-k.o. and C57BL/6J groups performed similarly to the vehicle-treated animals of each respected group (*data not shown*).

## Discussion

Uncontrolled pain remains a public health epidemic, with countless people suffering from a multitude of disorders that cost society billions of dollars annually. The American Pain Society recently released a statement indicating that translational approaches to pain relief (*i.e., *bench to bedside) would have "immediate and profound benefits" [31]. Despite significant advances made to our understanding of molecular pain mechanisms, few novel analgesic therapies have managed to reach clinical practice [32]. This failure to translate from bench to beside is in part due to the use of inefficient behavioral assays in animal models of pain. Many assays typically require that the investigator both apply the pain stimulus and evaluate a reflexive response by the animal. This can be both time consuming and highly subjective. The use of these assays to model and quantify pain in animals is a major bottle-neck in the development of new analgesics, and providing obstacles in the validation and optimization of clinical treatment strategies.

The use of mice as the preferred model for pain testing provides the opportunity to utilize genetically-altered (i.e., knock-out) strains to study specific targets related to pain processing. The drawback of behaviorally testing mice relates to difficulties in producing fast and reliable results using an animal known for being skittish and jumpy. In this study, we have overcome these traits using our operant test paradigm by presenting a system whereby the investigator is removed from the field and the animal tests itself. We build on our prior work completed in rat models of orofacial pain [17,19,27].

As part of our battery of behavioral assessment assays, we evaluate the effects of a variety of factors (e.g., drugs) on general activity to determine if these factors could have an impact on the ability of an animal to perform the operant task. For example, we previously demonstrated that doses of morphine (≥ 2.5 mg/kg) produced significant reduction of rearing behavior in rats [27]. Another factor that may affect performance relates to inherent differences between strains of animals, as some strains may be relatively more active or inquisitive. Therefore, prior to testing the different mice strains (SKH1-Hr^hr^, C57BL/6J, TRPV1 k.o.) on the operant device, we evaluated them using the rearing assay. We found that the general activity for these three strains was not so much a factor, as they all had similar responses. In fact the rearing duration/event outcome was virtually identical for each strain by the end of two week acclimation period. We concluded that the general activity or exploratory behavior differences between these strains of mice would not likely influence their ability to complete the operant task. The rearing data indicates a potential habituation to the environment for all strains over the two-week test schedule, with each strain displaying decreased events and duration. However, in contrast, we found that the operant results improved with each session; indicating that motivation and cognitive factors are encouraging the animal to complete the task.

When we decided to modify the existing rat operant unit to test mice, we searched for a mouse that was equivalent to the hairless Sprague-Dawley (S.D.) rat that we routinely use and found that the hairless SKH1-Hr^hr ^mouse strain. While the SKH1-Hr^hr ^mouse is typically used in dermatological studies, it is not the typical strain used in pain studies. Here we demonstrated that this strain indeed follows a normal thermal stimulus response, as compared to the wild-type C57BL/6J mice. Additionally, they respond in the appropriate fashion following capsaicin-induced pain with decreased operant licking behavior, and then with morphine-rescue, with return of licking events to baseline levels in the presence of capsaicin. As demonstrated in Figure 3, they are quick to learn the task, therefore minimizing training time. We find this hairless strain to be extremely docile and easy to handle, more convenient to use, as we do not need to shave them in order to test them. The video clip provides a real-time demonstration of the ease of using these animals. Note how quickly the animals are independently and successfully completing the task once placed in the box. Given these traits, the SKH1-Hr^hr ^mice are certainly appropriate and ideal for use in future pharmacological studies.

Trigeminal nociceptors project to the nucleus caudalis and synapse with second-order neurons in the superficial layers, which are organized in the same way as the dorsal horn of the spinal cord, with the nucleus caudalis being laminated like the dorsal horn in spinal cord [33-36]. In fact, the nucleus caudalis extends and merges with the spinal dorsal horn in the cervical spinal cord [37] and has been termed the medullary dorsal horn [38]. Neurons within this superficial region have been shown to respond to cooling, cold, warming, and hot stimuli [39-42] and chemical irritants [42,43]. As it is considered a correlate to the spinal cord dorsal horn, the trigeminal spinal nucleus and trigeminal sensory system provides a relevant region with respect to the understanding of pain mechanisms in general [44,45]. Given this, we targeted this region using the ultrapotent TRPV1 agonist, RTX, in order to assess changes in orofacial pain.

We found that w.t. C57BL/6J mice treated with RTX had a significant decrease in the ability to sense hot (48°C) and very hot (55°C) stimulus temperatures. This contrasts the response of the TRPV1 k.o. mice, as they were only significantly affected at the hottest (55°C) temperature. These results appear to be inconsistent with the work completed by Caterina, et al., as they demonstrated a significant difference at high temperature stimuli, such as 55°C on the hot plate assay, when comparing the TRPV1 k.o. versus the w.t. mice [46]. This is interesting because the thermal assay difference between the reflex and operant tasks may explain this discrepancy. For example, for the reflex-based hot-plate assay, the difference between the k.o. and w.t. groups may be a function of the wild-type mice being more sensitive to a rapid temperature increase, so the response may be a function of the temperature difference detection plus the thermal pain producing the response. In addition, when within-group effects were evaluated for temperature, there was a decreased latency for both the TRPV1 k.o. and w.t. mice. While these authors did not report on this comparison, there appears to be a significant decrease in hot plate latency in the TRPV1 k.o. group as the stimulus moves to higher temperatures. This is consistent with what we found using the operant test, with a significant decrease in licking outcome at the 55°C stimulus. Collectively, these results indicate that while RTX is specific for the TRPV1 receptor, other receptors co-expressed (e.g., TRPV2) on the same neurons with TRPV1 may be susceptible to the RTX-lesioning effects within the nucleus caudalis. Another possibility is that a population of A-δ fibers expressing TRPV1 is also lesioned following RTX-treatment in the w.t. mice.

When we evaluated the pain sensitivity of these mice using an unlearned-reflexive measure such as the capsaicin eye-wipe assay, the RTX-treated C57BL/6J mice appeared to respond identically as the TRPV1 k.o. mice. This brings up an interesting observation that this particular type of assay is sufficient for evaluating a gross-impairment in the nociceptive signaling pathway. However, it cannot tease out subtle differences uncovered using the operant assay, such as the different responses of these two groups at 55°C. This additional information may be relevant for the development of novel analgesics.

In conclusion, we have successfully used the operant orofacial assay to evaluate and characterize thermal pain sensitivity in mice, thus providing a revolutionary step in the ability to model and study pain. Pain is ultimately experienced as a culmination of complex information from the periphery (including the location, intensity, quality, and time course). While *in vitro *studies can provide insight into the individual components, adequate behavioral assessment of animal models is requisite for understanding the integration of these components into the perception of pain. These findings demonstrate that operant methods, which reflect physiological and cerebral processing of pain, can provide insights not possible with reflex-based testing alone. Such insight may enhance our understanding of pain and ultimately lead to the ability to treat uncontrollable. This replicates what occurs with humans, whereby a person may need to choose between tolerating pain in order to receive some reward (*e.g., *going to a nice dinner with a migraine headache versus not going to a bad restaurant with the same type of headache). As pain spans any number of diseases, ranging from diabetes to cancer, providing a means of quickly identifying new analgesic agents would provide a tremendous societal benefit, and this mouse operant system provides such a way.

## Competing interests

The authors declare that they have no competing interests.

## Authors' contributions

JN conceived and participated in the design and supervision of the study, analyzed the data and drafted the manuscript. CK was involved with the behavioral testing and writing portions of the manuscript. WM and JW were involved with behavioral testing. FW, AJ, and HR were involved with the design of the study and behavioral testing. RC was involved with the design of the study and interpretation of the data. All authors read and approved the final manuscript.

## Supplementary Material

Additional file 1 Movie showing a hairless SKH1-Hr^hr ^mouse testing itself on the operant orofacial device. This movie demonstrates the ease of testing a mouse in an investigator-independent fashion using the operant orofacial device. Note the technician placing the mouse in the testing box at the beginning of the video – this is the last time the technician interacts with the animal during the trial. At ~16 sec, the animal initiates the first successful contact with the thermode and reward bottle. We pan to the computer at ~31 sec to illustrate the online recordings (top trace = reward licking signal; bottom trace = stimulus thermode signal), and at ~43 sec, we demonstrate how the uncompressed, high-speed acquisition signal appears. Please note that each peak on the top trace represents a single lick. Click here for file
